# Structural and Functional Shifts in the Microbial Community of a Heavy Metal-Contaminated Soil Exposed to Short-Term Changes in Air Temperature, Soil Moisture and UV Radiation

**DOI:** 10.3390/genes15010107

**Published:** 2024-01-16

**Authors:** Isabel Silva, Marta Alves, Catarina Malheiro, Ana Rita R. Silva, Susana Loureiro, Isabel Henriques, M. Nazaret González-Alcaraz

**Affiliations:** 1 CEF (Center for Functional Ecology), Associate Laboratory TERRA, Department of Life Sciences, Faculty of Sciences and Technology, University of Coimbra, 3000-456 Coimbra, Portugal; silva.isabel@ua.pt; 2 CESAM (Centre for Marine and Environmental Studies), Department of Biology, University of Aveiro, 3810-193 Aveiro, Portugal; catarinalemosmalheiro@ua.pt (C.M.); ritas@ua.pt (A.R.R.S.); sloureiro@ua.pt (S.L.); 3 CBQF (Center for Biotechnology and Fine Chemistry), School of Biotechnology, Portuguese Catholic University, 4169-005 Porto, Portugal; msalves@ucp.pt; 4 Department of Agricultural Engineering of the E.T.S.I.A., Technical University of Cartagena, 30203 Cartagena, Spain

**Keywords:** climate change, increased temperature, soil drought, soil flood, UVR exposure, soil pollution, soil invertebrates, *Enchytraeus crypticus*, soil microbiome, metagenomics

## Abstract

The interplay between metal contamination and climate change may exacerbate the negative impact on the soil microbiome and, consequently, on soil health and ecosystem services. We assessed the response of the microbial community of a heavy metal-contaminated soil when exposed to short-term (48 h) variations in air temperature, soil humidity or ultraviolet (UV) radiation in the absence and presence of *Enchytraeus crypticus* (soil invertebrate). Each of the climate scenarios simulated significantly altered at least one of the microbial parameters measured. Irrespective of the presence or absence of invertebrates, the effects were particularly marked upon exposure to increased air temperature and alterations in soil moisture levels (drought and flood scenarios). The observed effects can be partly explained by significant alterations in soil properties such as pH, dissolved organic carbon, and water-extractable heavy metals, which were observed for all scenarios in comparison to standard conditions. The occurrence of invertebrates mitigated some of the impacts observed on the soil microbial community, particularly in bacterial abundance, richness, diversity, and metabolic activity. Our findings emphasize the importance of considering the interplay between climate change, anthropogenic pressures, and soil biotic components to assess the impact of climate change on terrestrial ecosystems and to develop and implement effective management strategies.

## 1. Introduction

Soil contamination is one of the most pressing concerns globally [1]. Anthropogenic activities such as mining, industry or agriculture have released and continued to release large amounts of contaminants to the environment. In Europe, for example, the total number of potentially contaminated soil sites is around 2.5 million, with heavy metals being among the most frequently found contaminants [2]. This situation raises particular concern due to heavy metal nonbiodegradability, ecotoxicity and accumulative behavior [3,4].

Climate change may exacerbate the negative effects of soil heavy metal contamination on terrestrial ecosystems [5,6,7,8]. Future climate projections predict not only an increase in air temperature and ultraviolet (UV) radiation exposure but also a higher risk of flash flood events and more severe and prolonged droughts [9,10]. On the one hand, alterations in climate conditions can directly affect soil functions [11,12,13,14], while, on the other hand, they can also modify soil properties and, consequently, influence the retention, (im)mobilization and bioavailability of heavy metals [6,7,15]. Both aspects can influence the functionality of the soil system, resulting in the so-called multistressor effects. Therefore, studying the effects of heavy metal soil contamination allied with forecasted climate conditions provides valuable information to anticipate which areas may be most problematic and apply sustainable soil management practices, which are essential to restore and maintain soil biodiversity and functionality, as highlighted by the EU Soil Strategy for 2030 [16].

Soil microbiome is responsible for key processes, such as balancing the concentration of different trace gases (e.g., CO_2_ and CH_4_) or regulating soil acidity and nutrient cycling [17]. Additionally, the interaction between soil microorganisms and heavy metals can affect, for instance, heavy metals oxidation state and (im)mobilization through organic metal binding [18,19], thus determining their bioavailability and ecotoxicity [19,20]. Bacteria can, for instance, produce siderophores—chelating compounds that bind to metals such as Cd, Pb, and Zn, resulting in the formation of bioprecipitates with lower bioavailability [19,20,21]. Also, by changing the heavy metal oxidation state, bacteria can decrease metal ecotoxicity [18,19]. This interaction between heavy metals and soil microorganisms has been exploited in bioremediation strategies [19]. However, previous studies demonstrated that heavy metal contamination can negatively impact the soil microbiome and, therefore, compromise these functions [4,22,23,24]. Additionally, the interaction of heavy metals with other soil biotic key components of terrestrial ecosystems, such as invertebrates, can influence the response of the soil microbial community, both to heavy metal contamination and climate change alterations [25]. In fact, it is known that invertebrates can accumulate heavy metals [26], changing their bioavailability in the soil. However, alterations in climate factors can also change the bioaccumulation of heavy metals in soil invertebrates [27,28]. Additionally, the presence of soil invertebrates can also have an impact on the soil microbiome structure, composition and metabolic activity [29]. By participating in nutrient cycling, soil invertebrates can potentially influence the microbial metabolic activity in the soil [30]. Also, invertebrate feeding, burrowing, and casting activities can alter soil structure and modify soil conditions, resulting in soil microhabitats that can support specific microorganisms, thereby influencing soil microbiome richness and diversity [31]. Nevertheless, as mentioned above, heavy metal contamination can also influence soil invertebrates and their activity in the soil and consequently change their influence on soil microbiome. 

The aim of the present study was to investigate how short-term changes in individual climate factors, namely air temperature, soil moisture, and UV emission, impact the microbial community in heavy metal-contaminated soil. Moreover, we aimed also to assess whether soil invertebrates could influence the microbial community’s response to the climate conditions simulated. For this, a heavy metal-contaminated soil was incubated for 48 h under different climate scenarios established according to the Intergovernmental Panel on Climate Change (IPCC) predictions for Europe by 2100 [9,10], without and with the presence of *E. crypticus*—a soft-bodied oligochaete with a key role in terrestrial ecosystems functioning by participating, for instance, in the biogeochemical cycling of organic matter and nutrients [32,33]. Effects on soil microbial community structure, composition and functions were evaluated by using culture-dependent and -independent analyses. The initial hypothesis posited that brief exposure to variations in air temperature, soil moisture and UV radiation would induce alterations in the activity, structure, composition, and functions of the microbial community within the heavy metal-contaminated soil. It was further hypothesized that the observed changes would depend on the simulated climate scenario and on the presence of soil invertebrates.

## 2. Materials and Methods

### 2.1. Heavy Metal-Contaminated Test Soil

The Chemical Complex of Estarreja (CCE) is one of the main industrial areas in Central-Northern Portugal (Figure S1, Supplementary Materials). For years, solid and liquid wastes containing high levels of potentially toxic elements (e.g., heavy metals) have been discharged and accumulated in nearby areas, including agricultural fields, urban areas, watercourses, and coastal wetlands [34,35]. Heavy metal inputs were mainly associated with the generation of sulfuric acid through arsenopyrite roasting (e.g., As, Cu, Ni, Pb, and Zn) and from a chlor-alkali facility (e.g., Hg) [34,36]. In addition to the industrial activity, the agricultural and livestock practices in the area have further contributed to the degradation of the local agroecosystems.

A former agricultural soil in the vicinity of the CCE affected by heavy metal contamination was selected to perform the study. The soil was collected from the top 5 cm, air dried, homogenized, sieved (2 mm mesh), completely characterized, and stored at 4 °C to minimize the biological activity. González-Alcaraz et al. (2019) have detailed the entire process of soil characterization [37] (Table S1, Supplementary Materials). The test soil showed a concentration of heavy metals above the background levels described for Portuguese soils [38] and, in some cases, above the reference values established by the Portuguese Environmental Agency for contaminated soils [39].

### 2.2. Soil Invertebrate Species

The soil invertebrate *E. crypticus* (phylum Annelida, class Oligochaeta, family Enchytraeidae) [40] was selected because it shows a high tolerance to different soil properties (e.g., texture, pH, organic matter) [41,42,43] and is increasingly used as a bioindicator of the response of the soil system to stress conditions (e.g., contamination, climate alterations) [7,33]. Sexually mature organisms, identifiable by the clearly visible clitellum, measuring approximately 1 cm in length and showing no visible problems, were used for the study. A complete description of the culture process is provided by González-Alcaraz et al. (2019) [37].

### 2.3. Climate Scenarios Simulated

The activities involving the transformation of C and N by soil microorganisms are typically evaluated by subjecting the soil to standardized climatic conditions. According to the OECD guidelines [44,45], these conditions include maintaining a constant air temperature of ≈20 °C, a steady soil moisture of approximately 50% of the maximum water holding capacity (WHC) of the soil, and no control of the UV radiation exposure. Taking these standard conditions as a starting point, a set of climate scenarios was established according to the IPCC projections for southern Europe for 2100 [9,10]. Simulations of these climate scenarios involved altering a single climate factor (air temperature, soil moisture, or UV radiation) while keeping the others at levels recommended by the OECD guidelines (Table 1; Figure S2, Supplementary Materials).

#### 2.3.1. Air Temperature

Two climate scenarios were simulated according to the air temperature rise predictions (Figure S2a, Supplementary Materials): (i) soil exposed to a daily air temperature regime between 15 °C and 25 °C; (ii) soil exposed to a daily air temperature between 20 °C and 30 °C. These scenarios involved cyclic air temperature patterns lasting 24 h, replicating both rises and falls in daily air temperature. Detailed information on the temperature regime cycles is available in the Supplementary Materials.

#### 2.3.2. Soil Moisture Content

Two climate scenarios were simulated based on predicted soil moisture changes (Figure S2b, Supplementary Materials): (i) soil moistened at 25% WHC (simulating decreased soil water availability during severe droughts; hereafter referred to as drought); (ii) soil moistened at 75% WHC (simulation of increased soil water availability after heavy rains and/or floods; referred to as flood).

#### 2.3.3. UV Radiation

One climate scenario was established considering the UV radiation exposure increase (Figure S2c, Supplementary Materials). This scenario comprised a recurring UV radiation cycle lasting 24 h (exposure to UV radiation for 6 h followed by 18 h without UV radiation) based on the UV radiation data available for Lisbon (i.e., the nearest city to the area where the heavy metal-contaminated soil was gathered with a comprehensive UV radiation record; https://www.temis.nl/index.php). The cycle was based on the minimum (≈7) and maximum (≈10) average values for the summer UV index (i.e., the season with the highest UV index) for the period 2002 and 2018. Detailed information on the UV radiation regime cycle and the exposure setup is available in the Supplementary Materials (Figure S3, Supplementary Materials).

### 2.4. Soil Incubations

The heavy metal-contaminated test soil (50 g of pre-moistened soil) underwent a 48 h incubation in 170 mL glass containers (9.5 cm height, 5 cm diameter) under the different climate scenarios simulated, including the standard conditions advised by the OECD guidelines (5 replicates). Each simulated climate scenario, as well as the standard conditions, were tested with and without *E. crypticus* (30 organisms per container initially placed on the soil surface following the recommendations of the standardized ISO and OECD guidelines to perform ecotoxicity tests with *Enchytraeus* sp. [46,47]). The test containers were covered with perforated parafilm and randomly positioned inside acclimatized rooms or chambers with a 16 h light and 8 h dark photoperiod. Air temperature simulations were conducted in a KBWF 720 Binder acclimatized chamber (Binder, Holzgerlingen, Germany), while soil moisture content and UV radiation simulations were performed in acclimatized rooms. Following a 48 h incubation period, soil samples were taken for physicochemical determinations, community-level physiological profiling and DNA extraction. Special care was taken to avoid sampling *E. crypticus* individuals along with the soil samples.

### 2.5. Physicochemical Soil Analysis

Fresh soil aliquots were used to measure soil moisture (dried at 65 °C until a constant weight was achieved) and stored at −20 °C prior to laboratory analyses. Soil:water mixtures (1:5 *w*:*v*, 2 h at 200 rpm) were prepared in a shaker with thawed samples (3 replicates). Suspensions were centrifuged (Megafuge 8R Centrifuge, 20 min at 4500 rpm and 4 °C), filtered (0.45 µm, Microsart CN-filter Sartorius, Goettingen, Germany) and analyzed for pH (WTW-pH 330i/set meter), electrical conductivity—EC (WTW 3110/set meter), dissolved organic carbon—DOC (TOC-VCSH Shimadzu, samples acidification with HCl purity 37%), and water-extractable heavy metals—MeW (Al, As, Cd, Cu, Fe, Mn, Pb, Sb and Zn; ICP-Ms Agilent 7500A, Agilent Technologies, Inc., Santa Clara, CA, USA; detection limit, d.l. ≤ 2 µg L^−1^; samples acidification before analysis with HNO_3_ purity 65%).

### 2.6. Microbiological Soil Analysis

#### 2.6.1. Physiological Profiling at the Community Level

The Biolog EcoPlate system (BIOLOG Inc., Hayward, CA, USA) was used to determine the physiological profiles [48]. Microbial catabolic activity was determined by calculating the average well color development (AWCD) following the method outlined by Samarajeewa et al. (2017) [48]. The preference for the consumption of specific C source groups (i.e., polymers, carbohydrates, carboxylic and acetic acids, amino acids, and amines and amides) was calculated as the substrate average well color development (SAWCD) [49]. For this, 3 g of fresh soil and 27 mL of sterile water were shaken with 20 sterile glass beads for 10 min at 200 rpm in an orbital shaker (5 replicates). The resulting mixture was then diluted to obtain a 100-fold working solution. EcoPlates, containing 100 μL of the working solution in each well, were incubated at 20 °C in the dark for 6 days. Every 24 h, the color development of each well was measured by determining the optical density (OD) at 590 nm using an automated plate reader (Biolog, MicroStation, Hayward, CA, USA).

#### 2.6.2. DNA Extraction and Quantitative PCR

Soil aliquots were frozen in liquid N and kept at −80 °C. The PureLink Microbiome DNA Purification Kit (Invitrogen, Thermo Fisher Scientific, Carlsbad, CA, USA) was used for total genomic DNA extraction of 0.25 g of soil following manufacturer instructions (3 replicates). To determine bacterial abundance, the 16S rRNA gene was measured using quantitative PCR (qPCR). For each soil DNA sample, three independent PCR reactions were prepared with primers 338F (CCT ACG GGA GGC AGC AG) and 518R (CCT ACG GGA GGC AGC AG) [50] in a CFX96™ Real-Time PCR System (Bio-Rad, Hercules, CA, USA). The reaction mixture (20 μL) consisted of 10 µL of Speedy NZYTaq 2× Green Master Mix (Nzytech, Lisboa, Portugal), 7.2 μL of sterilized water, 0.4 μL of each primer at 10 µM, and 2 μL of DNA template. The amplification conditions included an initial step of 95 °C for 7 min, and 30 amplification cycles of 95 °C for 15 s and 65 °C for 30 s. Melting analysis was performed from 55 °C to 95 °C, with steady 0.1 °C increments at each 5 s. The analysis of DNA was conducted utilizing the standard curve method detailed in Brankatschk et al. (2012) [51].

#### 2.6.3. Illumina High-Throughput Sequencing

The microbiome profiling was executed by Eurofins Genomics with MiSeq (Ebersberg, Germany). For each sample of DNA, the hypervariable V3–V4 region of the 16S rRNA gene was amplified, and the resulting sequencing data were analyzed following Eurofins Genomics protocols. Briefly, reads were demultiplexed, followed by merging using FLASH software (2.2.00) [52]. Merged reads were quality-filtered to remove reads with less than 285 bp, ambiguous bases (“N”) and sequences with an average quality lower than Q30. Chimeric reads were then identified and removed using the UCHIME algorithm in the VSEARCH software package (Version 2.13.1) [53]. To identify operational taxonomic units (OTUs), high-quality reads were processed using Minimum Entropy Decomposition (MED) [54]. Taxonomy assignment was performed by DC-MEGABLAST alignments of representative cluster sequences to the NCBI database (Release 2019-10-10). For sequences to be considered, a minimal requirement was a sequence identity of 70% across at least 80% of the representative sequence [55]. Subsequent processing of OTUs and taxonomic assignments was carried out using the QIIME software package (Version 1.9.1, http://qiime.org/, accessed on 1 July 2019). To enhance estimates, the abundance of bacterial taxonomic units was normalized using lineage-specific copy numbers of the marker genes [56]. The nucleotide sequences were deposited in GenBank under the accession numbers SAMN33218491 to SAMN33218526.

#### 2.6.4. Inference of Bacterial Community Function from 16S rRNA Gene Sequencing Data

Piphillin software [57] was used to predict functions for soil microbial populations. The OTU sequences and an OTU abundance table were used to match OTUs to the Kyoto Encyclopedia of Genes and Genomes database of phylogenetically referenced prokaryotic genomes (KEGG; http://www.genome.jp/kegg/ accessed on 1 July 2019). For that, an identity cutoff of 97% was used to obtain a list of KEGG orthologs (KO) and their abundance for each sample. 

### 2.7. Statistical Analysis

Statistical analyses were conducted with IBM SPSS Statistics 26 (IBM Corporation, Endicott, NY, USA); differences were considered significant at *p* < 0.05. Levene’s and Shapiro-Wilk were applied to check for normal distribution and equal variance, respectively. To discern differences between the standard conditions (i.e., conditions advised by OECD guidelines) and the climate scenarios simulated (increased air temperature, drought, or flood conditions, and increasing UV radiation), one-way ANOVA followed by Dunnett’s post hoc test was conducted. Analysis was done separately for incubations without and with *E. crypticus* since each approach had its own control (soil incubations under standard conditions without invertebrates in the first case and soil incubations under standard conditions with invertebrates in the second case).

PRIMER v6 software (Primer-E Ltd., Plymouth, UK) was applied to conduct clustering analysis and principal coordinate analysis (PCoA), based on a Bray–Curtis distance matrix calculated after the OTU abundance table was square root transformed. Differences in bacterial communities’ structure after exposure to each climate scenario were assessed using PERMANOVA, followed by the pairwise Monte Carlo (MC) test (999 unrestricted permutations). Species richness (number of observed OTUs) and diversity (Shannon–Wiener index) were obtained using the diversity function from the vegan package [58] of R software version 4.0.2 [54]. Canonical correlation analyses (CCA) between microbial diversity (OTU abundance table) and physicochemical soil characteristics (environmental data) were performed using the cca function from the vegan package [58] in R [59]. Environmental variables that did not follow the CCA normal distribution assumption were removed from the analysis. This was checked using mshapiro.test function of the package mvnormtest in R [60]. Multicollinearity between environmental variables was verified using cor and pairs functions in R [59]. Based on this, all variables with a correlation above 0.94 were removed from the analysis. The influence of each environmental variable and their interaction on the cumulative canonical eigenvalues was examined through one-way ANOVA using the aov function of R [59].

## 3. Results

### 3.1. Changes in Physicochemical Soil Characteristics

Irrespective of the presence of soil invertebrates, at least one of the physicochemical soil parameters measured was affected by the short-term exposure to each climate scenario tested, compared to standard conditions (Tables S2 and S3, Supplementary Materials). For soil without *E. crypticus* (Table S2, Supplementary Materials), the exposure to increased air temperatures and flood conditions led to a statistically significant increase in pH values (from 5.7 under standard conditions to 5.8–5.9). The exposure to drought was the only climate scenario that led to a significant decrease in soil pH (to 5.6). All the climate scenarios simulated significantly decreased the concentration of Cd_W_ (from 15.8 μg kg^−^^1^ under standard conditions to 0.7–4.9 µg kg^−^^1^). On the other hand, the exposure to flood conditions led to a significant increase in the levels of As_W_ (from 5138 to 18,662 µg kg^−1^) and Mn_W_ (from 338 to 615 µg kg^−^^1^), compared to standard conditions. For soil with *E. crypticus* (Table S3, Supplementary Materials), the exposure to increased air temperatures and drought conditions led to significant increases in pH values (from 5.7 under standard conditions to 5.8–5.9). Drought also induced a significant decrease in EC (from 0.05 to 0.03 dS m^−^^1^), Cu_W_ (from 712 to 403 µg kg^−^^1^), FeW (from 1546 to 822 µg kg^−^^1^) and Sb_W_ (from 128 to 79 µg kg^−^^1^) compared to standard conditions. On the contrary, after 48 h under flood conditions, significant increases were observed in the concentration of As_W_ and Mn_W_ (from 5195 to 21,726 µg kg^−^^1^ for As and from 340 to 589 µg kg^−^^1^ for Mn), while UV radiation exposure led to an increase in Al_W_ and Pb_W_ (from 1738 to 3272 µg kg^−^^1^ for Al and from 217 to 476 µg kg^−^^1^ for Pb).

### 3.2. Changes in Soil Community-Level Physiological Profiles

**Figure 1:**
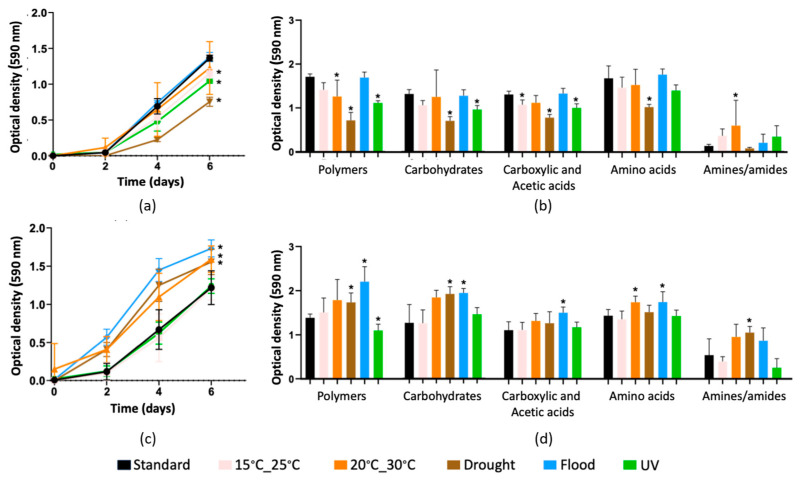
Physiological profiles of the microbial community in heavy metal-contaminated soil under distinct climate scenarios, as indicated by average well color development (AWCD, (**a**,**c**)) and substrate average well color development (SAWCD, (**b**,**d**)). Exposure tests were conducted without (**a**,**b**) and with (**c**,**d**) *E. crypticus*. Microbial catabolic activity was measured over a 6-day period following 48 h of exposure to the different scenarios. (**b**,**d**) Substrate consumption at the end of the 6-day period. The values are presented as averages ± SD (n = 5), with the standard denoting the climate conditions advised by the OECD guidelines. An asterisk (*) indicates statistical differences compared to standard conditions based on one-way ANOVA followed by Dunnett’s post hoc test (*p* ≤ 0.05). UV (ultraviolet).

Substrate consumption rate (AWCD) showed a 1–2 d delay before metabolic reactions began to take place (Figure 1). This was more evident in the absence of soil invertebrates. After that, AWCD increased with time and attained peak values after 6 d of incubation. For soil without *E. crypticus*, the exposure to drought conditions led to the lowest AWCD, with a 45% significant decrease compared to standard conditions (Figure 1a). A significant decrease was also registered for soil exposed to UV radiation (−23%) and to air temperatures between 15 °C and 25 °C (−16%). Drought conditions significantly decreased the oxidation of polymers (−58%), carbohydrates (−47%), carboxylic and acetic acids (−41%), and amino acids (−38%) (inferred from SAWCD; Figure 1b); UV exposure decreased the oxidation of polymers (−35%), carbohydrates (−24%), and carboxylic and acetic acids (−24%); and air temperatures between 15 °C and 25 °C decreased the oxidation of carboxylic and acetic acids (−19%). On the contrary, the presence of *E. crypticus* led to significant increases in AWCD compared to standard conditions after exposure to flood (+42%), air temperatures between 20 °C and 30 °C (+31%), and drought (+28%) (Figure 1c). The significantly affected substrates were polymers (+59% with flood, +26% with drought, and −21% with UV exposure), carbohydrates (+59% with flood and +52% with drought), carboxylic and acetic acids (+36% with flood), amino acids (+22% with flood and +22% with 20 °C–30 °C), and amine/amides (+93% with drought) (Figure 1d).

### 3.3. Changes in Soil Bacterial Abundance, Diversity, and Community Structure

**Figure 2:**
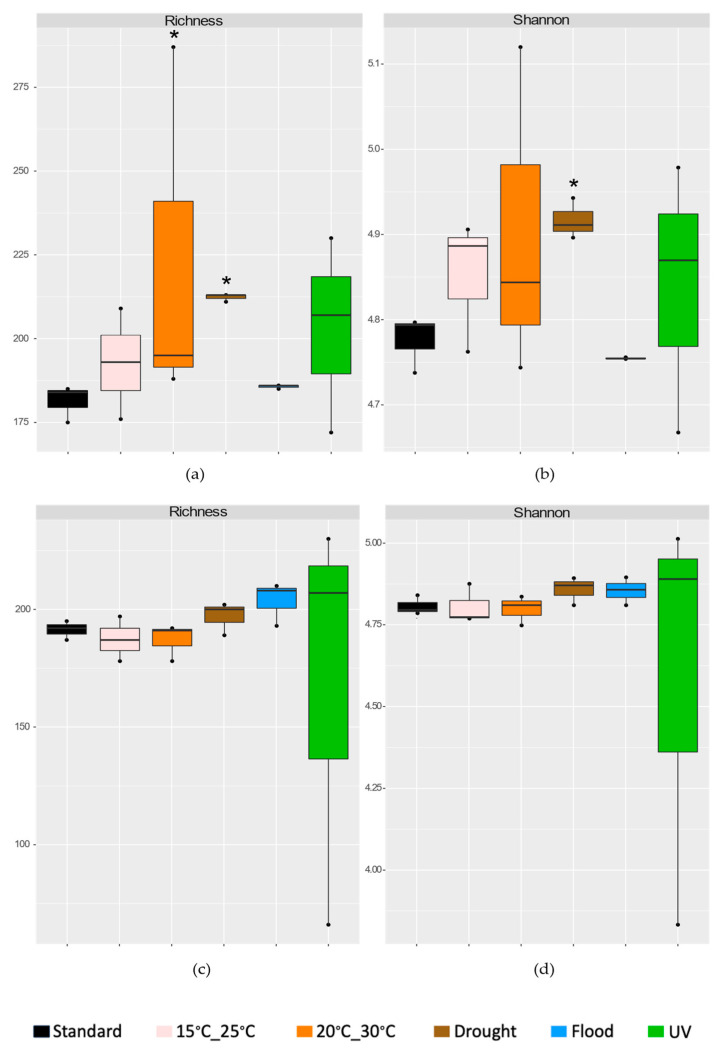
Measurements of α diversity encompassing richness (**a**,**c**) and Shannon index (**b**,**d**), utilizing 16S rRNA gene profiling of the heavy metal-contaminated soil after a 48 h exposure to each simulated climate scenario, both in the absence (**a**,**b**) and the presence (**c**,**d**) of *E. crypticus.* Continuous lines within the boxes show the median value (n = 3). The boxes include data within the 25th and 75th percentiles, while the whisker lines indicate the 5th and 95th percentiles. Standard climate conditions are the ones recommended by OECD. Asterisk (*) denotes statistically significant differences in comparison to the standard condition, employing one-way ANOVA and Dunnett’s post hoc test (*p* ≤ 0.05). UV (ultraviolet).

**Figure 3:**
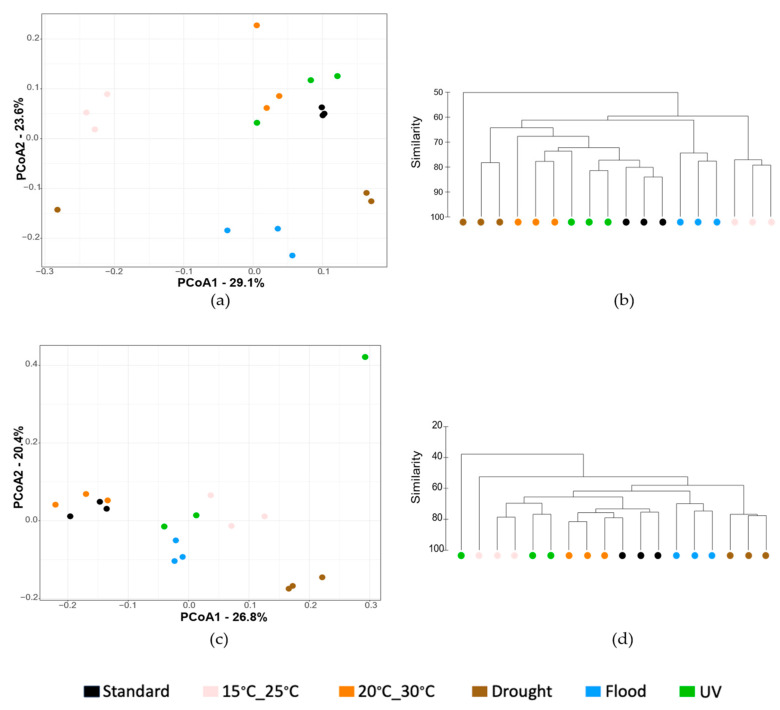
β diversity of the heavy metal-contaminated test soil following a 48 h exposure to simulated climate scenarios, both in the absence (**a**,**b**) and the presence (**c**,**d**) of *E. crypticus*. Principal coordinate analysis (PCoA, (**a**,**c**)) and dendrograms (**b**,**d**) were constructed using Bray–Curtis similarity matrices derived from OTUs abundance tables. Standard refers to the climate conditions advised by OECD. UV (ultraviolet).

In terms of bacterial abundance (Figure S4, Supplementary Materials), the only significant effect was observed when the soil was exposed to UV radiation without *E. crypticus* (−53% copies of 16S rRNA gene compared to standard conditions). In terms of α diversity (Figure 2), significant effects were only registered when the test soil without *E. crypticus* was exposed to air temperatures between 20 °C and 30 °C (+23% in richness) and drought conditions (+17% in richness and +3% in diversity) (Figure 2a,b). There were no significant changes in terms of abundance and α diversity indexes in any of the climate scenarios tested in the presence of invertebrates (Figure 2c,d). In terms of bacterial community structure, without *E. crypticus*, a significant impact was observed when the soil was exposed to air temperatures between 15 °C and 25 °C (*p* = 0.013) and flood conditions (*p* = 0.009) (Figure 3a,b). The PCoA exposed a separation between communities exposed to these climate scenarios and the ones exposed to standard conditions, sharing with the latter 62% and 64% of similarity, respectively. With *E. crypticus*, a significative impact on bacterial community structure resulted from exposure to air temperatures between 15 °C and 25 °C (*p* = 0.04), and drought and flood conditions (*p* = 0.01 and *p* = 0.02, respectively) (Figure 3c,d). These communities shared 59%, 54% and 61% similarity with the ones from standard conditions, respectively.

**Figure 4:**
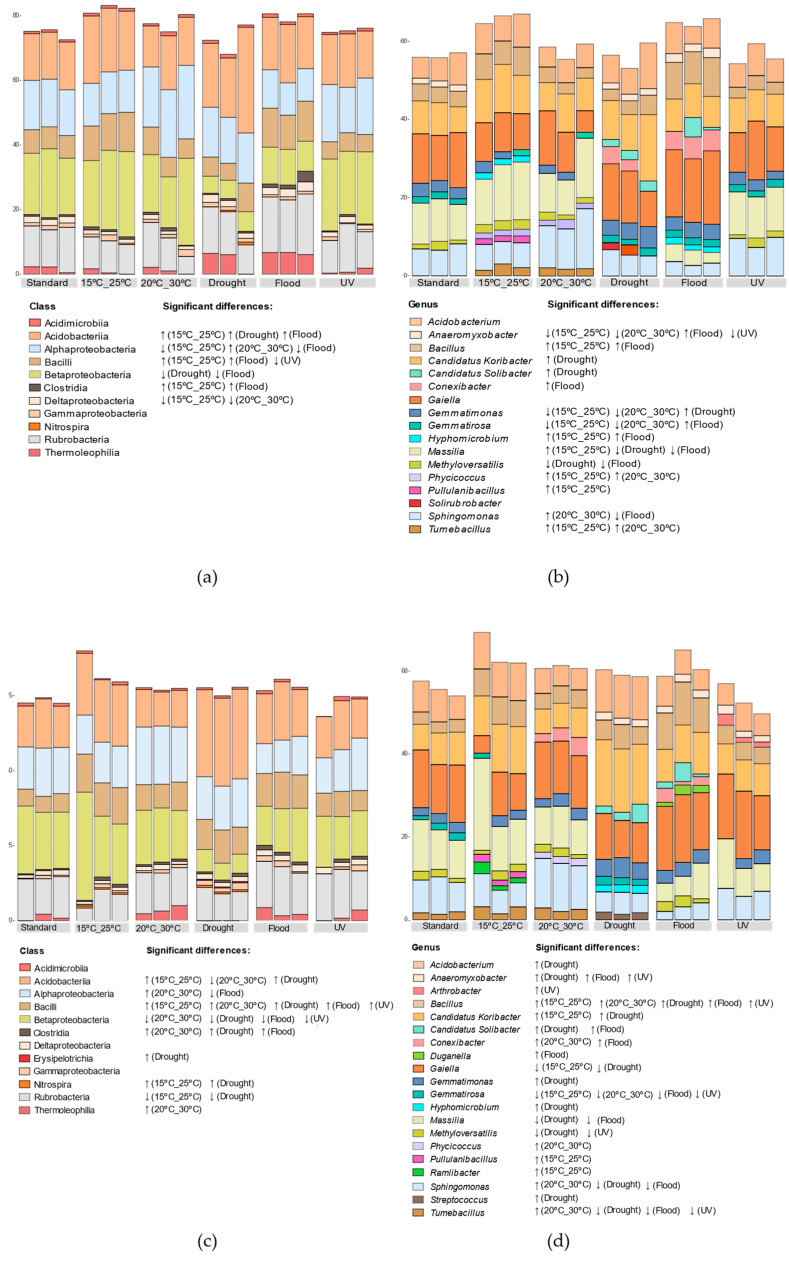
Variation in bacterial classes’ relative abundance (**a**,**c**) and the top 15 most prevalent genera (**b**,**d**) in the heavy metal-contaminated following a 48 h exposure to the climate conditions, both in the absence (**a**,**b**) and in the presence (**c**,**d**) of *E. crypticus*. The findings of 3 replicates are provided. Standard designates the climate conditions advised by OECD. Arrows indicate significant differences (↓ decrease; ↑ increase) compared to standard conditions (determined by one-way ANOVA followed by Dunnett’s post hoc test; *p* < 0.05). UV (ultraviolet).

All the climate scenarios simulated induced a significant change in the relative abundance of at least one bacterial class, compared to the standard conditions. In the absence of *E. crypticus*, the scenarios with the greatest impact were flood conditions and air temperatures between 15 °C and 25 °C, each with a significant impact on the relative abundance of five classes (Figure 4a). Acidobacteriia, Bacilli and Clostridia increased significantly under these two climate scenarios, while that of Alphaproteobacteria decreased. In the presence of *E. crypticus*, on average, the number of affected bacterial classes in each simulated climate scenario was higher compared to standard conditions (Figure 4c). For example, under air temperatures between 20 °C and 30 °C and drought conditions, six classes were significantly affected. The most frequently affected classes were Bacilli (increased in all climate scenarios simulated relative to standard conditions) and Betaproteobacteria (decreased in all climate scenarios except at 15 °C–25 °C). At the genus level, without *E. crypticus*, climate scenarios that simulated increased air temperatures led to the strongest shifts (Figure 4b). For example, the exposure to temperatures between 15 °C and 25 °C increased the relative abundance of *Bacillus*, *Hyphomicrobium*, *Massilia*, *Phycicoccus*, *Pullulanibacillus* and *Tumebacillus*; and the exposure to temperatures between 20 °C and 30°C increased the relative abundance of *Phycicoccus*, *Sphingomonas* and *Tumebacillus*. Both temperature regimes led to a decrease in *Anaeromyxobacter*, *Gemmatimonas* and *Gemmatirosa*. With *E. crypticus*, drought conditions were the climate scenario that led to the strongest changes (Figure 4d), increasing the relative abundance of *Acidobacterium*, *Anaeromyxobacter*, *Bacillus*, *Candidatus Koribacter*, *Candidatus Solibacter*, *Gemmatimonas*, *Hyphomicrobium* and *Streptococcus* in soil; and decreasing *Gaiella*, *Massilia*, *Methyloversatilis*, *Sphingomonas* and *Tumebacillus*.

**Figure 5:**
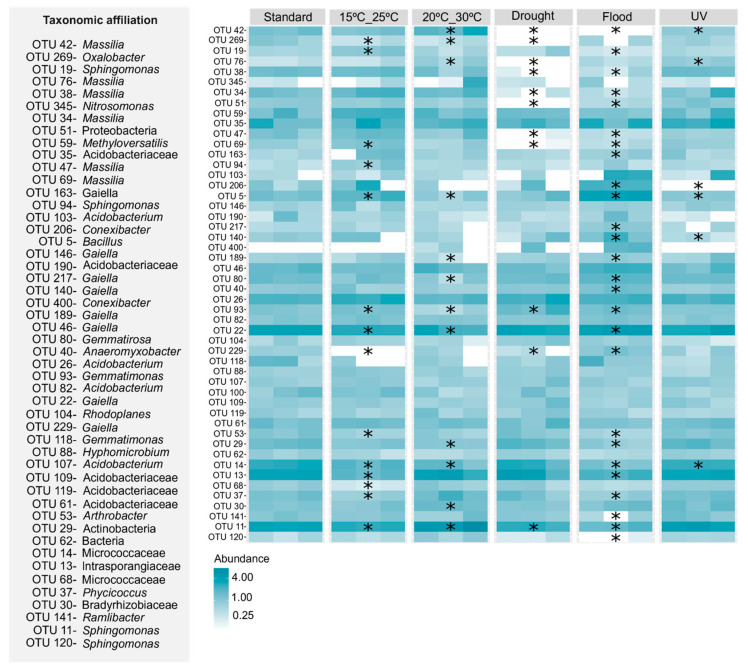
Heatmap showing the most prevalent OTUs in the heavy metal-contaminated test soil (top 30 per treatment) following a 48 h exposure to each simulated climate scenario, excluding the presence of *E. crypticus*. The color spectrum reflects the relative abundance of OTUs in each soil replicate (n = 3). Standard refers to climate conditions advised by OECD. Asterisk (*) indicates statistically significant differences compared to standard conditions (one-way ANOVA followed by Dunnett’s post hoc test (*p* ≤ 0.05)). UV (ultraviolet).

**Figure 6:**
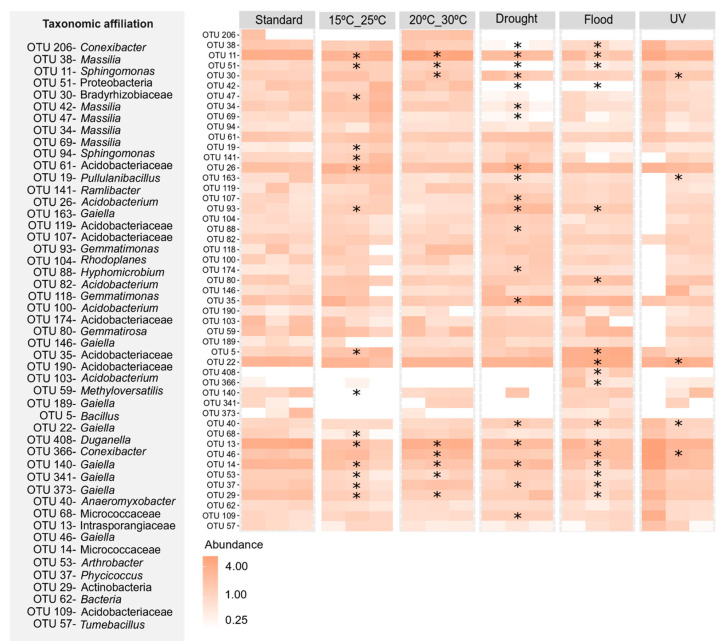
Heatmap showing the most prevalent OTUs in the heavy metal-contaminated test soil (top 30 per treatment) following a 48 h exposure to each simulated climate scenario with *E. crypticus*. The color spectrum reflects the relative abundance of OTUs in each soil replicate (n = 3). Standard refers to climate conditions recommended by OECD. Asterisk (*) indicates statistically significant differences compared to standard conditions (one-way ANOVA followed by Dunnett’s post hoc test (*p* ≤ 0.05)). UV (ultraviolet).

At the OTU level, the exposure to flood conditions was the scenario that induced the greatest changes in the heavy metal-contaminated soil without *E. crypticus* (Figure 5), significantly affecting 52% of the top 30 most abundant OTUs. The increase in soil moisture content decreased 58% of these OTUs that belonged to *Sphingomonas*, *Massilia Phycicoccus*, *Ramlibacter*, *Arthrobacter* and *Pullulanibacillus* genera. When *E. crypticus* was present in the soil, the exposure to drought conditions was the scenario that caused the strongest impact at the OTU level (Figure 6), affecting 39% of the top 30 most abundant OTUs. The decrease in soil moisture content decreased 53% of these OTUs that belonged to *Sphingomonas*, *Massilia*, *Phycicoccus* and *Gaiella* genera.

### 3.4. Predicted Functional Pathways Alterations

Without *E. crypticus*, 26% of the top 30 most abundant bacterial functions in the soil were predicted to be significantly affected compared to standard conditions by the exposure to air temperatures between 15 °C and 25 °C (Table S4, Supplementary Materials). A significant decrease in pathways related with carbohydrate metabolism (i.e., pyruvate and propanoate metabolism); and a significant increase in signal transduction (two-component system) and cell motility (flagellar assembly) related pathways were predicted. In the case of exposure to alterations in soil moisture content, 19% (flood) and 16% (drought) of these functions were predicted to be significantly affected. The biosynthesis of amino acids, particularly the cysteine and methionine metabolism, and the carbon fixation pathway were predicted to undergo an increase under flood conditions; while functions related to the nucleotide metabolism (purine metabolism), carbohydrate metabolism (glyoxylate and dicarboxylate metabolism) and xenobiotic degradation (benzoate degradation) were predicted to suffer a decrease (Table S4, Supplementary Materials). Under drought conditions, the biosynthesis of amino acids and the 2-oxocarboxylic acid metabolism were predicted to be positively affected, while translation (aminoacyl-tRNA biosynthesis) and cell motility (flagellar assembly) related pathways were predicted to suffer a decrease. With *E. crypticus*, 42% of the top 30 most abundant bacterial functions in the soil were predicted to be significantly affected compared to standard conditions by exposure to the flood scenario (Table S5, Supplementary Materials). This was the case of fatty acid metabolism and several carbohydrate metabolism-related pathways (i.e., pyruvate and butanoate metabolism), for which a significant decrease was predicted after the exposure to flood conditions. On the contrary, the oxidative phosphorylation was predicted to be positively affected. In the presence of invertebrates, 39%, 26% and 6% of the most abundant bacterial functions in the soil were predicted to be affected after exposure to increased air temperatures (15 °C–25 °C and 20 °C–30 °C) and drought conditions, respectively (Table S5, Supplementary Materials). After exposure to 15 °C–25 °C, a significant decrease was predicted for the fatty acid metabolism and several carbohydrate metabolism-related pathways (i.e., pyruvate and propanoate metabolism), while an increase was predicted for signal transduction-related functions (i.e., two-component system). Most of the functions inferred to be the most abundant in soil were predicted to be negatively affected by drought (i.e., purine, pyruvate, pyrimidine, glyoxylate and dicarboxylate metabolism and benzoate degradation pathways).

### 3.5. Relative Contribution of Physicochemical Soil Characteristics to Bacterial Community Structure

**Figure 7:**
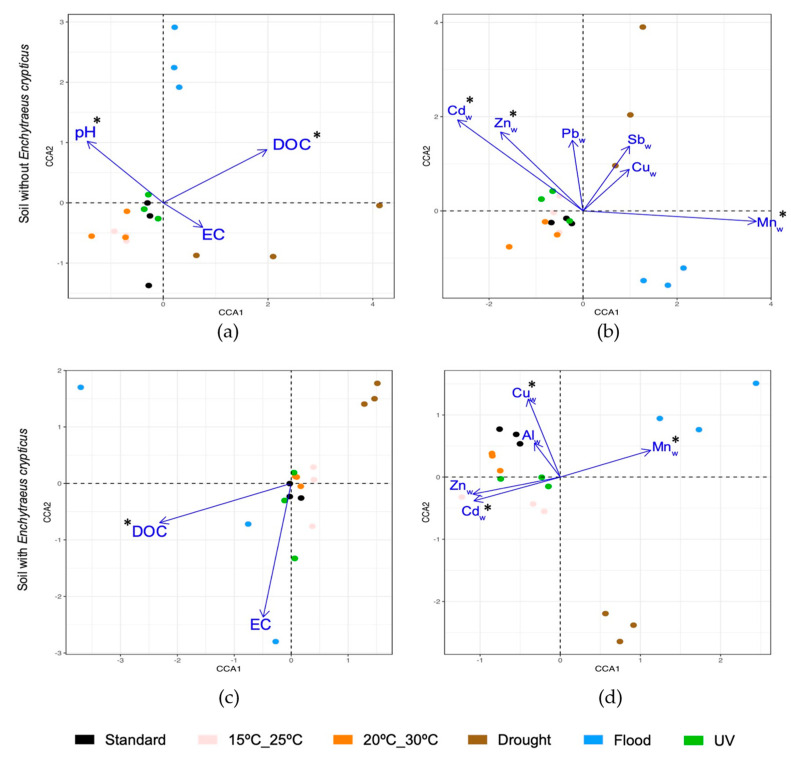
Canonical correspondence analysis (CCA) for the heavy metal-contaminated test soil after exposure for 48 h to each of the climate scenarios without (**a**,**b**) and with (**c**,**d**) the presence of the invertebrate *E. crypticus*. Ordination diagram of the bacterial community data together with soil variables: pH, electrical conductivity (EC), dissolved organic carbon (DOC), and water-extractable heavy metal concentrations (Me_W_). For soil without invertebrates CCA1 and CCA2 each explained: 46.9% and 32.1% (**a**); and 35.2% and 21.6% (**b**). For soil with invertebrates CCA1 and CCA2 each explained: 59.8% and 40.2% (**c**); and 33.9% and 25% (**d**). Standard refers to the climate conditions recommended by the OECD guidelines. Asterisk (*) indicates significant drivers of changes in bacterial community structure based on ANOVA (*p* ≤ 0.05).

Canonical correspondence analysis (CCA) was used to investigate the contribution of different physicochemical soil parameters to the changes observed in bacterial community structure after short-term exposure to the different climate scenarios simulated (Figure 7). In the absence of *E. crypticus*, the CCA results indicated that soil pH (*p* = 0.03), DOC (*p* = 0.01), Cd_W_ (*p* = 0.02), Zn_W_ (*p* = 0.03) and Mn_W_ (*p* = 0.01) were significant drivers of changes in bacterial community structure, particularly when the soil was exposed to drought and flood conditions (Figure 7a,b). When the soil was incubated in the presence of *E. crypticus*, the CCA results showed that the soil bacterial community structure was particularly affected by changes in DOC (*p* = 0.03), Cd_W_ (*p* = 0.02), Cu_W_ (*p* = 0.01) and Mn_W_ (*p* < 0.01). Again, communities exposed to drought or flood conditions were particularly affected by these parameters (Figure 7c,d).

## 4. Discussion

It is increasingly evident that changes in climate conditions have and will have important implications for the composition and functioning of terrestrial ecosystems. Among others, the soil microbiome is one of the ecosystem components that is likely to be most affected. When these changing climate conditions are combined with the presence of contaminants, such as heavy metals, the scenario that arises is even more complex given the combination of stress factors with which soil microorganisms must deal. Although the possible effects of climate change alterations should be deduced from medium/long-term studies, short-term exposures to changing climate conditions, such as those tested in the present study, also provide relevant information on the response of soils to extreme and occasional weather events. The latter can complement medium/long-term observations but also help to provide early warning tools to further improve the management of contaminated areas.

Regarding soil microbial catabolic activity, in the absence of invertebrates, three of the five climate scenarios simulated induced a significant decrease compared to standard conditions (i.e., without climate stress for soil microorganisms). The highest decrease was observed after exposure to soil drought, which could be related to the lower solubilization of most of the substrates used by bacteria and/or the lower availability of nutrients due to water scarcity [61,62]. This result agrees with a previous study in which a similar experimental setup was used but with noncontaminated soil [29], as well as with longer exposure times studies [63]. To a lower extent, the exposure to UV radiation and air temperatures between 15 °C and 25 °C also led to a decrease in the soil microbial catabolic activity when the test soil was incubated without invertebrates. In the case of the UV radiation exposure scenario, the drop in the microbial catabolic activity coincided with a significant decrease in total bacterial abundance (estimated from the quantification of the 16S rRNA gene). Although soil surface bacteria are normally resistant to UV radiation [64,65], the abundance decrease may be the result of the interaction of UV radiation with the heavy metals present in the soil. It is known that UV radiation has significant effects on the chemical speciation of metals such as Cu, Fe, and Mn, all present in the test soil, possibly increasing metal ecotoxicity [66]. By contrast, the exposure to flood conditions was the only scenario that did not decrease the microbial catabolic activity of the test soil in the absence of invertebrates, which could reflect the increase in soil DOC after 48 h exposure (although not significant with respect to standard conditions). This may be associated with the observed increase in functions related to carbon fixation pathways, which convert inorganic carbon into organic compounds [67]. Differently, in the presence of invertebrates, a significant increase in the soil microbial catabolic activity was observed after exposure of the test soil to flood, drought and air temperatures between 20 °C and 30 °C compared to standard conditions, with higher utilization rates of, for example, polymers, carbohydrates and amino acids. These results suggest that the presence of soil invertebrates could attenuate the negative effects of the climate scenarios simulated on the soil microbial community, allowing the maintenance of its activity. This effect was previously observed for noncontaminated soil [29] and could be attributed to the key role that invertebrates play in modulating soil porosity and compaction, favoring the oxygenation of the medium and the access to water and nutrients [25,68,69], thus benefiting microbial communities. Moreover, the possible ingestion and accumulation of heavy metals by soil invertebrates could contribute to the attenuation effect. Soil invertebrates like *E. crypticus* can accumulate heavy metals [27,70], decreasing their availability to the soil microbial community. However, due to the short exposure time of this study (48 h), the amount of heavy metals ingested was probably negligible. Nevertheless, previous studies showed that climate alterations can change heavy metal bioaccumulation patterns in soil invertebrates [8,27], which could compromise the beneficial functions of these organisms for the soil microbiome.

Regarding the structure and composition of the soil bacterial community, all the climate scenarios simulated had an impact on the heavy metal-contaminated test soil with respect to standard conditions. Furthermore, the latter differed depending on the absence or presence of invertebrates during short-term exposures. Without invertebrates, exposure to soil flood and increased air temperatures, mainly to 15 °C–25 °C, were the climate scenarios that resulted in the greatest impact at the class level. These two scenarios decreased the relative abundance of classes belonging to Proteobacteria (Alphaproteobacteria, Betaproteobacteria and Deltaproteobacteria) and increased those belonging to Firmicutes (Bacilli and Clostridia). The decrease in the relative abundance of Alphaproteobacteria and Deltaproteobacteria differed from what was observed in a previous study performed with noncontaminated soil [29]. Firmicutes are normally more resistant to heavy metal contamination and heavy metal shifts in the soil, while Proteobacteria are more sensitive to these changes [3]. Considering this, the decrease in the relative abundance of classes belonging to Proteobacteria observed in this study could be related to climate–heavy metal interactions [71]. The class Acidobacteriia was also affected in the absence of invertebrates, increasing its relative abundance after exposure to increased air temperatures and drought and flood conditions. In the case of drought, the increase in the Acidobacteriia class, whose members prefer acidic conditions [72,73,74], was accompanied by a decrease in soil pH. Soil pH is one of the primary factors driving the distribution and function of soil microorganisms, the effect of which outweighs, in some cases, that of nutrient availability [17,75,76]. In fact, the CCA analysis suggests that, without invertebrates, soil pH was a significant driver of bacterial community structural changes, particularly after exposure to drought conditions.

Besides soil pH, in the absence of invertebrates, the CCA analysis also suggests that DOC was another significant driver of bacterial community structural changes, particularly after exposure to flood conditions (positive correlation between DOC in the soil and bacterial communities exposed to flood). Changes in DOC concentration can directly affect the reduction and oxidation of heavy metals in the soil, contributing to the transformation of these heavy metals into a more toxic form [77]. It is known, for instance, that a flood situation may reduce As(V) to the more toxic form As(III) [71]. Additionally, the increase in soil moisture content is normally associated with an increase in heavy metals bioavailability and, consequently, in their ecotoxicity in soil [71]. This was the case of As and Mn, whose concentrations in water significantly increased after exposure to flood conditions. This was also observed by González-Alcaraz et al. (2019) [37] after the short-term incubation of heavy metal-contaminated soils to different moisture regimes. The above observations could be an explanation for the changes registered for the soil microbial community after exposure to flood conditions. In fact, this climate scenario was the one that had the highest impact on the soil microbial community at the OTU level, affecting 52% of the most abundant OTUs present in the soil and decreasing OTUs affiliated to genera with important functions in soils such as *Massilia* and *Sphingomonas* [78,79].

The presence of the invertebrate altered the response of the microbial community of the heavy metal-contaminated test soil to the different climate scenarios simulated in terms of its structure, composition, and functions. In this case, increased air temperatures, mainly 20 °C–30 °C, and drought conditions were the climate scenarios that had the greatest impact on soil bacterial classes, resulting in lower relative abundance of Betaproteobacteria and higher of Bacilli. With invertebrates, as also observed in the short-term exposures without them, drought and flood conditions decreased the abundance of genera with important functions for the soil [78,79]. *Massilia*, with a significantly lower relative abundance after exposure to soil drought or flood, have been described as playing important functions in plant growth promotion and reduction of nitrates in soils [74]. Also, *Sphingomonas*, capable of decomposing persistent organic contaminants and fixing atmospheric nitrogen in soils [79], decreased after exposure to these scenarios. The CCA analysis suggests that in the presence of invertebrates, DOC and heavy metal concentrations in water were significant drivers of bacterial community structural changes, particularly after exposure to drought and flood conditions. 

Both in the presence or absence of the invertebrate, the predicted functional changes were consistent with the observed changes in community structure but may also be related with short-term adaptations to distinct climate scenarios. For instance, in the case of a temperature increase to 15 °C–25 °C, a decrease in pathways related with carbohydrate metabolism may be part of a strategy to reduce heat generation [80]. Conversely, an increase in functions related to stress response, such as signal transduction pathways and cell mobility, was observed for this and other scenarios, both in the presence or absence of *E. crypticus*. In flood and drought conditions, the alterations in genes associated with amino acid biosynthesis may be linked to osmoregulation strategies, where these compounds play a crucial role [81]. Flood conditions appear to have stimulated the proliferation of autotrophic microorganisms, consistent with the predicted increase in genes related to carbon fixation, as mentioned earlier. Results showed a decrease in genes related to xenobiotic degradation under flood and drought conditions, which may also be associated with changes in community structure. In the case of flood scenarios, a decrease in the concentration of these compounds is expected due to increased water content. Xenobiotic degradation pathways play an important role in removing aromatic compounds from the soil [82]. Many contaminated sites are affected both by heavy metals and aromatic compounds, contaminants of particular concern due to their ecotoxicity [83]. In drought conditions, the predicted functional changes seem to be related with strategies to avoid water loss and desiccation, evidenced by a reduction in cell mobility and protein synthesis, for example [84].

Although all the climate scenarios simulated had an impact on soil bacterial community composition, changes in both bacterial richness and diversity were only observed after the exposure of the test soil to drought conditions in the absence of invertebrates (a significant increase compared to standard conditions). This could be related to a possible fragmentation of the medium because of the reduced soil moisture content, which could contribute to increasing the richness and diversity of the bacterial community by providing more isolated microhabitats formed by soil aggregates, which could allow different bacteria to grow [85,86,87]. On the contrary, in the presence of invertebrates, no changes were observed in the richness and diversity of the soil microbial community after exposure to drought conditions. This also highlights the possible buffering effect that the presence of invertebrates could have during the short-term incubations, attenuating the impact of the climate scenarios simulated on the microbiome of the heavy metal-contaminated test soil.

## 5. Conclusions

Short-term alterations in single climate factors (air temperature, soil moisture content, and UV radiation) had significant consequences on the microbial community activity, structure, composition, and functions of heavy metal-contaminated soil. The climate scenarios that induced the greatest impact were increased air temperatures and alterations in soil moisture content, simulating higher (flood) or lower (drought) soil water availability. The effects can be, at least in part, explained by the impact of the climate scenarios simulated on some soil parameters such as pH, DOC, and water-extractable heavy metal concentrations, which, in turn, may affect heavy metals bioavailability and ecotoxicity. The presence of soil invertebrates attenuated some of the observed effects induced by the climate scenarios simulated on the soil microbial community. This was particularly the case for microbial metabolic activity that decreased upon exposure to most of the climate scenarios simulated in the absence of invertebrates, while it increased in the presence of invertebrates. In future studies, this result should be further elucidated through the incorporation of RNA-seq data. Significant impacts on bacterial abundance, richness and diversity were also only observed in the absence of invertebrates.

These results highlight the importance of considering microorganisms–invertebrates–heavy metals interactions when assessing the impact of climate change alterations on the microbial communities of heavy metal-contaminated soils. Given the number of contaminated soils worldwide, the combined effects of heavy metal contamination and climate change alteration may dramatically restrict the ability of these systems to provide key services on which our societies and economies critically depend. Standardized conditions of air temperature, soil moisture content and light are therefore of low relevance when studying the microbiome in natural soils, underpredicting potential effects or changes in communities.

## Figures and Tables

**Table 1:** Climate scenarios simulated during the 48 h of incubation of soil samples without and with *E. crypticus*. Standard conditions denote the climate conditions advised by OECD guidelines. WHC (water holding capacity). UV (ultraviolet).

Climate Scenario	Climate Factor		
	Air Temperature (°C)	Soil Moisture (% WHC)	UV Radiation
Standard conditions	20	50	Without
Air temperature	15–25	50	Without
	20–30	50	Without
Soil moisture	20	25	Without
	20	75	Without
UV radiation	20	50	With

## Data Availability

The data presented in this study are available in article and Supplementary Material.

## Supplementary Materials

The following supporting information can be downloaded at: https://www.mdpi.com/article/10.3390/genes15010107/s1, Figure S1: Location map of the test soil sampling site; Table S1: Characterization of the heavy metal-contaminated test soil; Figure S2: Visual representation of the scenarios simulated: (a) air temperature; (b) soil moisture content; (c) ultraviolet (UV) radiation. Figure S3: Experimental set-up for (UV) radiation scenario; Table S2: pH, electrical conductivity (EC), dissolved organic carbon (DOC) and water-extractable heavy metals concentration (Me_W_) in the test soil after exposure to the different climate scenarios simulated without the presence of the invertebrate; Table S3: pH, electrical conductivity (EC), dissolved organic carbon (DOC) and water-extractable heavy metals concentration (Me_W_) in the test soil after exposure to the different climate scenarios simulated with the presence of the invertebrate; Figure S4: Absolute abundance of 16S rRNA gene in the heavy metal-contaminated test soil after exposure to each of the climate scenarios simulated for 48 h without and with the presence of the invertebrate; Table S4: Predicted functional pathway changes of the top 30 most abundant functions of the heavy metal-contaminated test soil after exposure to each of the climate scenarios simulated without the presence of the invertebrate; Table S5: Predicted functional pathway changes of the top 30 most abundant functions of the heavy metal-contaminated test soil after exposure to each of the climate scenarios simulated with the presence of the invertebrate [37,88,89,90,91].
