# Supplementary material for: Structural and Functional Shifts in the Microbial Community of a Heavy Metal-Contaminated Soil Exposed to Short-Term Changes in Air Temperature, Soil Moisture and UV Radiation
Source: Genes (Basel). 2024 Jan 16;15(1):107. doi: 10.3390/genes15010107 (PMC10815596; doi:10.3390/genes15010107)
Supplement: Supplementary file 1 [file genes-15-00107-s001.zip › genes-2808445-supplementary.pdf]

Supplementary Material

Structural and functional shifts in the microbial community of a heavy metal-contaminated soil exposed to short-term changes in air temperature, soil moisture and UV radiation

Isabel Silva[a,b], Marta Alves[c], Catarina Malheiro[b], Ana Rita R. Silva[b], Susana Loureiro[b], Isabel Henriques [a*], and M. Nazaret González-Alcaraz [b,d*]

[a]CEF (Center for Functional Ecology), Associate Laboratory TERRA & Department of Life Sciences, Faculty of Sciences and Technology, University of Coimbra, 3000-456 Coimbra, Portugal.

[b]CESAM (Centre for Marine and Environmental Studies) & Department of Biology, University of Aveiro, 3810-193 Aveiro, Portugal.

[c]CBQF (Center for Biotechnology and Fine Chemistry), School of Biotechnology, Portuguese Catholic University, 4169-005 Porto, Portugal.

[d]Department of Agricultural Engineering of the E.T.S.I.A., Technical University of Cartagena, 30203 Cartagena, Spain.

* Corresponding authors: isabel.henriques@uc.pt; nazaret.gonzalez@upct.es

Supplementary Material summary: 1 cover page, 2 text pages, 4 figures and 5 tables (in order of appearance in the article).

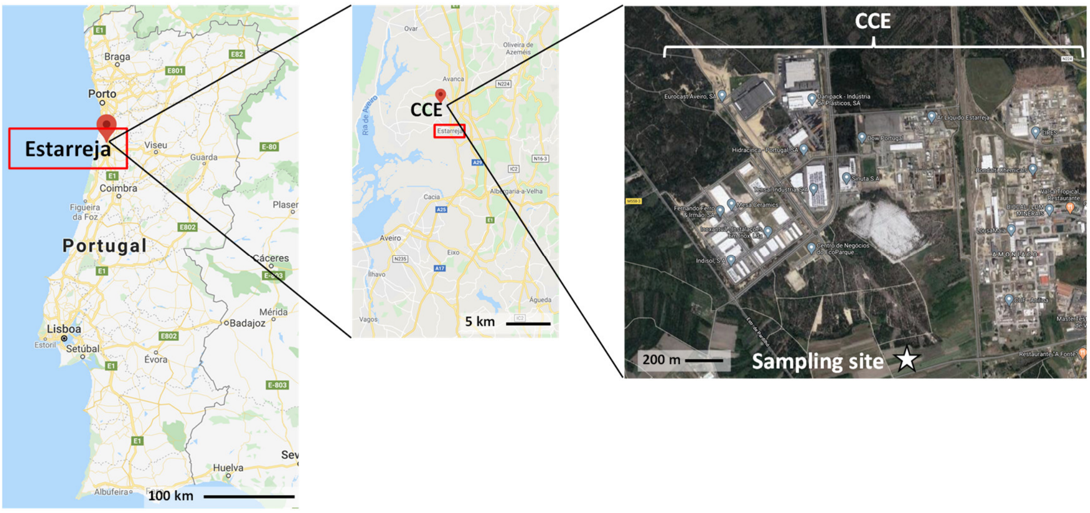

Figure S1. Location map of the test soil sampling site (40°46′04.7″N 8°34′59.0″W) in the vicinity of the Chemical Complex of Estarreja (CCE) in central-northern Portugal.

Table S1. Characterization of the heavy metal-contaminated test soil from central-northern Portugal (data retrieved from González-Alcaraz et al., 2019). Values are average ± SD (n=3). EC (electrical conductivity). TOC (total organic carbon). TN (total nitrogen). DOC (dissolved organic carbon). TDN (total dissolved nitrogen). CEC (cation exchange capacity). WHC (water holding capacity). Heavy metal $_T$ (heavy metal total concentration). Heavy metal $_W$ (heavy metal water-extractable concentration). d.l. (detection limit).

**Parameter**		**Parameter**		**Parameter**	
Texture[a]	Loamy sand				
Particle size distribution (%)[a]	Sand – 86.0 ± 0.0<br>Silt – 8.0 ± 0.0<br>Clay – 6.0 ± 0.0	Al_T (mg kg^{-1})[g]	70,603 ± 7490	Al_W (μg kg^{-1})[h]	1570 ± 269
pH-H_2O[b]	5.62 ± 0.09	As_T (mg kg^{-1})[g]	3087 ± 968	As_W (μg kg^{-1})[h]	6228 ± 146
EC (dS m^{-1})[b]	0.06 ± 0.001	Cd_T (mg kg^{-1})[g]	<d.l.	Cd_W (μg kg^{-1})[h]	<d.l.
TOC (mg kg^{-1})[c]	26.0 ± 1.4	Cu_T (mg kg^{-1})[g]	753 ± 204	Cu_W (μg kg^{-1})[h]	548 ± 16
TN (mg kg^{-1})[c]	1.65 ± 0.25	Fe_T (mg kg^{-1})[g]	34,613 ± 8035	Fe_W (μg kg^{-1})[h]	1508 ± 466
DOC (mg kg^{-1})[d]	125 ± 2	Mn_T (mg kg^{-1})[g]	375 ± 87	Mn_W (μg kg^{-1})[h]	633 ± 122
TDN (mg kg^{-1})[d]	22.3 ± 2.3	Ni_T (mg kg^{-1})[g]	35.3 ± 6.1	Ni_W (μg kg^{-1})[h]	70 ± 5.2
CEC ($cmol_c$ kg^{-1})[e]	7.8 ± 0.7	Pb_T (mg kg^{-1})[g]	1480 ± 419	Pb_W (μg kg^{-1})[h]	150 ± 59
WHC (%)[f]	36.5 ± 0.9	Sb_T (mg kg^{-1})[g]	<d.l.	Sb_W (μg kg^{-1})[h]	139 ± 2
		Zn_T (mg kg^{-1})[g]	977 ± 287	Zn_W (μg kg^{-1})[h]	1517 ± 46

[a] Bouyouco's densimeter method (Gee and Bauder, 1986).
[b] 1:5 (w:v) soil:H_2O suspensions.
[c] Elemental analyzer (LECO CHN628) (ISO, 1995).
[d] 1:5 (w:v) soil:H_2O suspensions and concentrations determined with TOC analyzer (TOC-VCSH Shimadzu).
[e] Saturation of soil exchange complex with 1N CH_3COONH_4 (Chapman, 1965).
[f] Soil saturation with water followed by water excess removal (ISO, 1998).
[g] X-ray fluorescence (Bruker S4 Pioneer).
[h] 1:5 (w:v) soil:H_2O suspensions and concentrations determined with ICP-MS (Agilent 7500A, Agilent Technologies, Inc., USA; detection limit, d.l. ≤2 μg L^{-1}).

References:

- Chapman, H.D. (1965). Cation Exchange Capacity. In: Black, C.A. (Ed.), Methods of Soil Analysis. American Society of Agronomy, Madison, pp. 891-901.

- Gee, G.W., & Bauder, J.W. (1986). Particle-size analysis. In: Klute, A. (Ed.), Methods of soil analysis. Part 1: Physical and Mineralogical Methods, second ed. American Society of Agronomy and Soil Science Society of America, Madison, United States, pp. 383-410.

- González-Alcaraz, M.N., Malheiro, C., Cardoso, D.N., & Loureiro, S. (2019). Soil moisture influences the avoidance behavior of invertebrate
species in anthropogenic heavy metal-contaminated soils. Environmental Pollution 248, 546-554.

- ISO (1995). ISO 10694: soil quality - determination of organic and total carbon after dry combustion (elementary analysis). ISO (Int. Org. Stand.). Geneva, Switzerland.

- ISO (1998). ISO 11274: soil quality - determination of the water-retention characteristics. ISO (Int. Org. Stand.). Geneva, Switzerland.

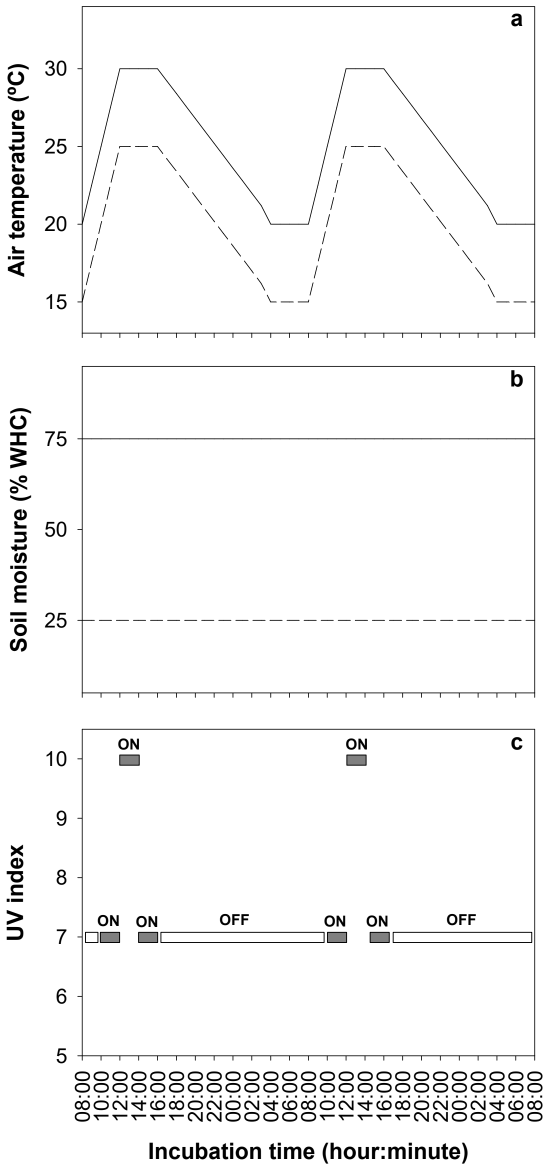

Figure S2. Climate scenarios simulated during the 48 h incubation of soil samples without and with the presence of the invertebrate *Enchytraeus crypticus*: a) air temperature (15 °C_25 °C and 20 °C_30 °C); b) soil moisture content (25% and 75% maximum soil water holding capacity, WHC); c) ultraviolet (UV) radiation (with UV radiation emission). In the UV radiation treatment "ON" indicates that the UV lamps were emitting UV radiation (in total 6 h per day) and "OFF" that there was no UV radiation emission (in total 18 h per day). For each climate factor modulated the

remaining factors were kept at the levels recommended by the standardized OECD guidelines (20 °C, 50% soil WHC, and no control of UV radiation).

Detailed description of the air temperature scenarios

The air temperature scenarios simulated started from the minimum air temperature (15 °C and 20 °C, respectively) at 8:00 and gradually increased until 12:00 when the maximum air temperature was reached (25 °C and 30 °C, respectively) and maintained until 16:00. From this time on, the air temperature gradually decreased until 4:00, when the minimum air temperature was reached again, and maintained until 8:00 to start a new air temperature regime cycle.

Detailed description of the ultraviolet (UV) radiation scenario and set-up

The UV radiation scenario simulated started without UV radiation emission from 8:00 to 10:00. At this time, UV radiation corresponding to a UV index of ≈7 started emitting until 12:00, when it was intensified up to ≈10. The UV index of ≈10 was maintained until 14:00, when it decreased again to ≈7 until 16:00. From this time on, the UV radiation emission ceased until 8:00 to start a new cycle of the UV radiation regime. In total, the soil was exposed to a daily UV dose of ≈4400 J m^{-2} (regular daily UV dose in Lisbon during the summer period). Ultraviolet (UV) radiation was provided by two UV lamps (Spectroline XX15F/B, Spectronics Corporation, USA) with peak emissions at 365 nm for UVA (≈320-400 nm) and 312 nm for UVB (≈280-320 nm). The UV lamps were covered by cellulose acetate sheets, previously burned

under the lamps for 12 h. This allowed the stabilization of the UV radiation intensity passing through and prevented the exposure of the test jars to UVC, which is not typically found in nature due to its strong adsorption to the stratosphere ozone layer. The different UV indexes selected were obtained by keeping the UV lamps in permanent, static position respect to fixed platforms where the test jars were located. One lamp was set at ≈75 cm distance from the test jars to reach the UV index of ≈7 and the other lamp at ≈65 cm distance to reach the UV index of ≈10. The test jars were moved between platforms to be exposed to the corresponding UV indexes and the perforated parafilm covers were removed during the periods of UV radiation emission. The UV radiation intensity reaching the test jars was checked with a spectroradiometer connected to a monochromator (Bentham Instruments, UK). The experimental set-up for the simulation of the UV radiation scenario is shown in Figure S3.

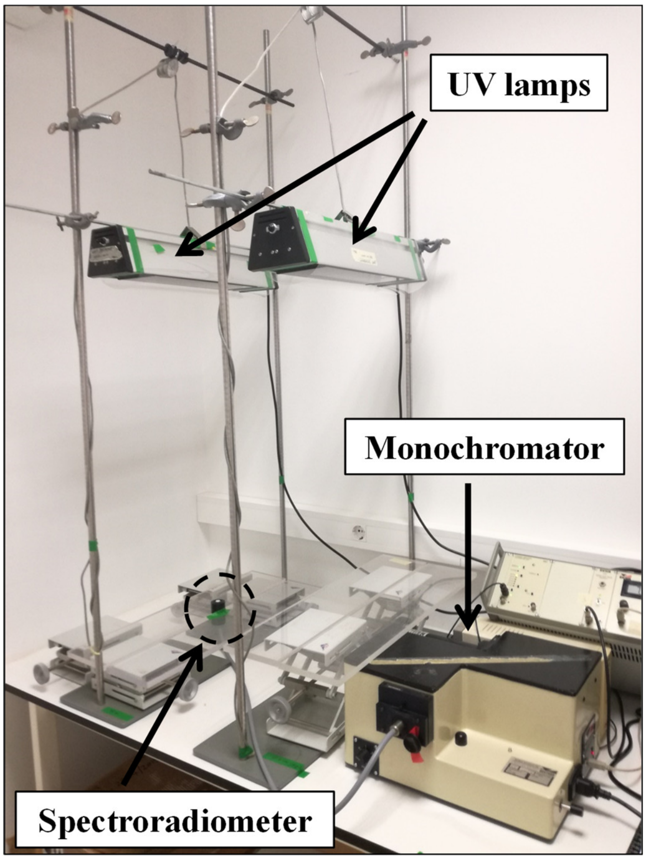

Figure S3. Experimental set-up for the simulation of the ultraviolet (UV) radiation scenario.

Table S2. pH, electrical conductivity (EC), dissolved organic carbon (DOC) and water-extractable heavy metal concentration (Me_W) in the test soil after exposure to the different climate scenarios simulated without the presence of the invertebrate *Enchytraeus crypticus*. Values are average ± SD (n=3). Standard refers to the climate conditions recommended by the OECD guidelines. Asterisk (*) indicates statistical differences in comparison to standard conditions (one-way ANOVA with Dunnett's post hoc test, $p \leq 0.05$). UV (ultraviolet).

Parameter	Standard	15 °C_25 °C	20 °C_30 °C	Drought	Flood	UV radiation
pH	5.71 ± 0.05	**5.87 ± 0.07***	**5.83 ± 0.02***	**5.59 ± 0.02***	**5.84 ± 0.01***	5.73 ± 0.01
EC (dS m^{-1})	0.05 ± 0.01	0.03 ± 0.005	0.04 ± 0.0002	0.06 ± 0.01	0.04 ± 0.001	0.05 ± 0.0005
DOC (mg kg^{-1})	60.6 ± 8.2	42.9 ± 6.5	47.4 ± 3.0	88.2 ± 22.4	76.3 ± 2.1	72.5 ± 4.7
Al_W (µg kg^{-1})	1,347 ± 994	1,634 ± 619	1,368 ± 104	2,044 ± 1,001	1,508 ± 401	3,072 ± 1,280
As_W (µg kg^{-1})	5,138 ± 1,183	4,521 ± 801	4,788 ± 212	5,262 ± 649	**18,662 ± 688***	5,647 ± 569
Cd_W (µg kg^{-1})	15.8 ± 4.2	**3.72 ± 0.90***	**3.93 ± 0.22***	**4.93 ± 1.83***	**0.67 ± 0.94***	**4.37 ± 0.73***
Cu_W (µg kg^{-1})	616 ± 224	519 ± 116	560 ± 24	651 ± 148	695 ± 25	738 ± 116
Fe_W (µg kg^{-1})	1,009 ± 658	1,464 ± 629	1,210 ± 116	2,014 ± 1,092	1,339 ± 292	3,161 ± 1,599
Mn_W (µg kg^{-1})	338 ± 126	293 ± 44	231 ± 19	397 ± 135	**615 ± 15***	352 ± 46
Pb_W (µg kg^{-1})	132 ± 104	201 ± 75	164 ± 15	249 ± 160	166 ± 33	434 ± 221
Sb_W (µg kg^{-1})	131± 31	89.2 ± 16.3	105 ± 7	125 ± 12	116± 9	129 ± 3
Zn_W (µg kg^{-1})	977 ± 463	970 ± 164	902 ± 32	894 ± 479	617 ± 119	1,384 ± 198

Table S3. pH, electrical conductivity (EC), dissolved organic carbon (DOC) and water- heavy metal concentration (Me_W) in the test soil after exposure to the different climate scenarios simulated with the presence of the invertebrate *Enchytraeus crypticus*. Values are average ± SD (n=3). Standard refers to the climate conditions recommended by the OECD guidelines. Asterisk (*) indicates statistical differences in comparison to standard conditions (one-way ANOVA with Dunnett's post hoc test, $p \leq 0.05$). UV (ultraviolet).

Parameter	Standard	15 °C_25 °C	20 °C_30 °C	Drought	Flood	UV radiation
pH	5.68 ± 0.02	**5.80 ± 0.04***	**5.85 ± 0.02***	**5.87 ± 0.03***	5.70 ± 0.03	5.67 ± 0.01
EC (dS m^{-1})	0.05 ± 0.002	0.05 ± 0.005	0.04 ± 0.0003	**0.03 ± 0.004***	0.05 ± 0.02	0.05 ± 0.004
DOC (mg kg^{-1})	65.8 ± 4.8	64.8 ± 6.0	60.2 ± 3.0	40.1 ± 6.8	98.8 ± 27.1	71.6 ± 4.4
Al_W (µg kg^{-1})	1,738 ± 427	1,379 ± 83	1,376 ± 194	1,063 ± 288	1,621 ± 554	**3,272 ± 475***
As_W (µg kg^{-1})	5,195 ± 1,060	5,529 ± 337	5,308 ± 89	3,269 ± 534	**21,726 ± 1,563***	5,704 ± 318
Cd_W (µg kg^{-1})	2.90 ± 0.57	4.73 ± 1.92	4.25 ± 1.04	3.05 ± 0.64	1.13 ± 1.60	5.73 ± 2.45
Cu_W (µg kg^{-1})	712 ± 106	655 ± 73	600 ± 9	**403 ± 76***	682 ± 80	740 ± 66
Fe_W (µg kg^{-1})	1,546 ± 202	1,110 ± 40	1,165 ± 131	**822 ± 207***	1,608 ± 543	3,369 ± 674
Mn_W (µg kg^{-1})	340 ± 146	377 ± 36	256 ± 8	335 ± 51	**589 ± 62***	354 ± 49
Pb_W (µg kg^{-1})	217 ± 40	148 ± 16	157 ± 12	118 ± 24	200 ± 72	**476 ± 101***
Sb_W (µg kg^{-1})	128 ± 20	116 ± 19	106 ± 3	**79 ± 138***	115 ± 10	126 ± 9
Zn_W (µg kg^{-1})	968 ± 507	1,164 ± 19	978 ± 38	838 ± 118	545 ± 201	1,300 ± 108

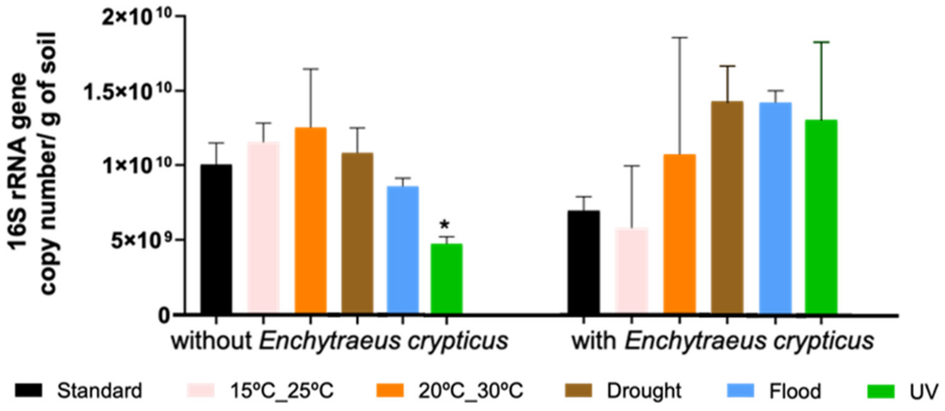

Figure S4. Absolute abundance of 16S rRNA gene in the heavy metal-contaminated test soil after exposure to each of the climate scenarios simulated for 48 h without and with the presence of the invertebrate *Enchytraeus crypticus*. Values are average ± SD (n=3). Standard refers to the climate conditions recommended by the OECD guidelines. Asterisk (*) indicates statistical differences in comparison to standard conditions based on one-way ANOVA followed by Dunnett's post hoc test ($p \leq 0.05$). UV (ultraviolet).

Table S4. Predicted functional pathway changes of the top 30 most abundant functions of the heavy metal-contaminated test soil after exposure to each of the climate scenarios simulated without the presence of the invertebrate *Enchytraeus crypticus*. Only significant differences in relative abundance towards the standard climate conditions (↓ decrease; ↑ increase) are indicated (one-way ANOVA followed by Dunnett's post hoc test, p<0.05).

Climate scenario	KEGG pathway	KEGG ID	Tendency	*p*
**15 °C_25 °C**	Microbial metabolism in diverse environments	ko01120	↓	0.05
	Two-component system	ko02020	↑	0.015
	Glyoxylate and dicarboxylate metabolism	ko00630	↓	0.05
	Pyruvate metabolism	ko00620	↓	0.05
	Propanoate metabolism	ko00640	↓	0.044
	Valine, leucine and isoleucine degradation	ko00280	↓	0.022
	Cysteine and methionine metabolism	ko00270	↑	0.043
	Flagellar assembly	ko02040	↑	0.012
**20 °C_30 °C**	Pyrimidine metabolism	ko00240	↓	0.05
**Drought**	Microbial metabolism in diverse environments	ko01120	↑	0.05
	Biosynthesis of amino acids	ko01230	↑	0.05
	Aminoacyl-tRNA biosynthesis	ko00970	↓	0.043
	2-Oxocarboxylic acid metabolism	ko01210	↑	0.002
	Flagellar assembly	ko02040	↓	0.018
**Flood**	Biosynthesis of amino acids	ko01230	↑	0.05
	Purine metabolism	ko00230	↓	0.05
	Glyoxylate and dicarboxylate metabolism	ko00630	↓	0.05
	Carbon fixation pathways in prokaryotes	ko00720	↑	0.05
	Benzoate degradation	ko00362	↓	0.017
	Cysteine and methionine metabolism	ko00270	↑	0.001

Table S5. Predicted functional pathway changes of the top 30 most abundant functions of the heavy metal-contaminated test soil after exposure to each of the climate scenarios simulated with the presence of the invertebrate *Enchytraeus crypticus*. Only significant differences in relative abundance towards the standard climate conditions (↓ decrease; ↑ increase) are indicated (one-way ANOVA followed by Dunnett's post hoc test, p<0.05).

Climate scenario	KEGG pathway	KEGG ID	Tendency	*p*
**15 °C_25 °C**	Microbial metabolism in diverse environments	ko01120	↓	0.002
	Biosynthesis of antibiotics	ko01130	↓	0.05
	Two-component system	ko02020	↑	0.05
	Glyoxylate and dicarboxylate metabolism	ko00630	↓	0.05
	Pyruvate metabolism	ko00620	↓	0.05
	Fatty acid metabolism	ko01212	↓	0.05
	Propanoate metabolism	ko00640	↓	0.05
	Valine, leucine and isoleucine degradation	ko00280	↓	0.05
	Porphyrin and chlorophyll metabolism	ko00860	↓	0.001
	2-Oxocarboxylic acid metabolism	ko01210	↑	0.05
	Alanine, aspartate and glutamate metabolism	ko00250	↓	0.05
	Flagellar assembly	ko02040	↑	0
**20 °C_30 °C**	Two-component system	ko02020	↑	0.05
	2-Oxocarboxylic acid metabolism	ko01210	↑	0.05
**Drought**	Purine metabolism	ko00230	↓	0.05
	Glyoxylate and dicarboxylate metabolism	ko00630	↓	0.05
	Pyruvate metabolism	ko00620	↓	0.05
	Pyrimidine metabolism	ko00240	↓	0.05
	Benzoate degradation	ko00362	↓	0.05
	Porphyrin and chlorophyll metabolism	ko00860	↓	0.031
	Cysteine and methionine metabolism	ko00270	↑	0.002
	2-Oxocarboxylic acid metabolism	ko01210	↑	0.05
**Flood**	Microbial metabolism in diverse environments	ko01120	↓	0.001
	Oxidative phosphorylation	ko00190	↑	0.009
	Glyoxylate and dicarboxylate metabolism	ko00630	↓	0.05
	Pyruvate metabolism	ko00620	↓	0.05
	Butanoate metabolism	ko00650	↓	0.012
	Amino sugar and nucleotide sugar metabolism	ko00520	↑	0.05
	Fatty acid metabolism	ko01212	↓	0.011
	Valine, leucine and isoleucine degradation	ko00280	↓	0.05
	Benzoate degradation	ko00362	↓	0.05
	Cysteine and methionine metabolism	ko00270	↑	0.004
	2-Oxocarboxylic acid metabolism	ko01210	↑	0.05
	Alanine, aspartate and glutamate metabolism	ko00250	↑	0.05
	Flagellar assembly	ko02040	↑	0.049
